# Notice of retraction

**DOI:** 10.1590/S1516-31802013000500014

**Published:** 2013-10-01

Notice is hereby given of the retraction of the article specified below. It has been retracted through the free initiative of the authors of the said article. The authors declare that this article constitutes reportage on recently published articles that haveappeared in the Brazilian scientific press and therefore does not contain any newly published results. It was written for the information of readers of São Paulo Medical Journal. It has been retracted because, as a consequence of an honest error, it was not prepared in observance of good practices for reviewing previously published literature. The retraction has been formally approved by the Editors of São Paulo Medical Journal.



**Retracted article:** Silva MR, Gomes AM. A review of recent pediatric research published in Brazilian indexed journals. Sao Paulo Med. J. 2012;130(5):318-29. doi:10.1590/S1516-31802012000500009.

São Paulo, August 2, 2013

**Authors:** Maurício Rocha e Silva and Ariane Maris Gomes

**Paulo Manuel Pego Fernandes** Editor, São Paulo Medical Journal

Os autores do artigo especificado declaram que retratam o mesmo por sua livre e espontânea vontade. Os autores declaram que este artigo constitui um relatório sobre artigos recentemente publicados na imprensa científica brasileira, de modo que não contém resultados científicos novos, com a finalidade específica de informar os leitores do São Paulo Medical Journal. O artigo é retratado porque, em virtude de um erro honesto, não foram observadas boas práticas para a escrita de uma revisão da literatura anteriormente publicada. Esta retratação é formalmente aceita pelos Editores do São Paulo Medical Journal.



**Artigo retratado:** Silva MR, Gomes AM. A review of recent pediatric research published in Brazilian indexed journals. Sao Paulo Med. J. 2012;130(5):318-29. doi:10.1590/S1516-31802012000500009.

São Paulo, 2 de agosto de 2013

**Authors:** Maurício Rocha e Silva e Ariane Maris Gomes

**Paulo Manuel Pego Fernandes** Editor, São Paulo Medical Journal

